# Validation of the Symptom Pattern Method for Analyzing Verbal Autopsy Data

**DOI:** 10.1371/journal.pmed.0040327

**Published:** 2007-11-20

**Authors:** Christopher J. L Murray, Alan D Lopez, Dennis M Feehan, Shanon T Peter, Gonghuan Yang

**Affiliations:** 1 Institute for Health Metrics and Evaluation, University of Washington, Seattle, Washington, United States of America; 2 School of Population Health, University of Queensland, Herston, Queensland, Australia; 3 Harvard Initiative for Global Health, Cambridge, Massachusetts, United States of America; 4 China Center for Disease Control and Prevention, Beijing, China; Umeå University, Sweden

## Abstract

**Background:**

Cause of death data are a critical input to formulating good public health policy. In the absence of reliable vital registration data, information collected after death from household members, called verbal autopsy (VA), is commonly used to study causes of death. VA data are usually analyzed by physician-coded verbal autopsy (PCVA). PCVA is expensive and its comparability across regions is questionable. Nearly all validation studies of PCVA have allowed physicians access to information collected from the household members' recall of medical records or contact with health services, thus exaggerating accuracy of PCVA in communities where few deaths had any interaction with the health system. In this study we develop and validate a statistical strategy for analyzing VA data that overcomes the limitations of PCVA.

**Methods and Findings:**

We propose and validate a method that combines the advantages of methods proposed by King and Lu, and Byass, which we term the symptom pattern (SP) method. The SP method uses two sources of VA data. First, it requires a dataset for which we know the true cause of death, but which need not be representative of the population of interest; this dataset might come from deaths that occur in a hospital. The SP method can then be applied to a second VA sample that is representative of the population of interest. From the hospital data we compute the properties of each symptom; that is, the probability of responding yes to each symptom, given the true cause of death. These symptom properties allow us first to estimate the population-level cause-specific mortality fractions (CSMFs), and to then use the CSMFs as an input in assigning a cause of death to each individual VA response. Finally, we use our individual cause-of-death assignments to refine our population-level CSMF estimates. The results from applying our method to data collected in China are promising. At the population level, SP estimates the CSMFs with 16% average relative error and 0.7% average absolute error, while PCVA results in 27% average relative error and 1.1% average absolute error. At the individual level, SP assigns the correct cause of death in 83% of the cases, while PCVA does so for 69% of the cases. We also compare the results of SP and PCVA when both methods have restricted access to the information from the medical record recall section of the VA instrument. At the population level, without medical record recall, the SP method estimates the CSMFs with 14% average relative error and 0.6% average absolute error, while PCVA results in 70% average relative error and 3.2% average absolute error. For individual estimates without medical record recall, SP assigns the correct cause of death in 78% of cases, while PCVA does so for 38% of cases.

**Conclusions:**

Our results from the data collected in China suggest that the SP method outperforms PCVA, both at the population and especially at the individual level. Further study is needed on additional VA datasets in order to continue validation of the method, and to understand how the symptom properties vary as a function of culture, language, and other factors. Our results also suggest that PCVA relies heavily on household recall of medical records and related information, limiting its applicability in low-resource settings. SP does not require that additional information to adequately estimate causes of death.

## Abstract 2

Chris Murray and colleagues propose and, using data from China, validate a new strategy for analyzing verbal autopsy data that combines the advantages of previous methods.

## Editors' Summary

**Background.:**

All countries need to know the leading causes of death among their people. Only with accurate cause-of-death data can their public-health officials and medical professionals develop relevant health policies and programs and monitor how they affect the nation's health. In developed countries, vital registration systems record specific causes of death that have been certified by doctors for most deaths. But, in developing countries, vital registration systems are rarely anywhere near complete, a situation that is unlikely to change in the near future. An approach that is being used increasingly to get information on the patterns of death in poor countries is “verbal autopsy” (VA). Trained personnel interview household members about the symptoms the deceased had before his/her death, and the circumstances surrounding the death, using a standard form. These forms are then reviewed by a doctor, who assigns a cause of death from a list of codes called the International Classification of Diseases. This process is called physician-coded verbal autopsy (PCVA).

**Why Was This Study Done?:**

PCVA is a costly, time-consuming way of analyzing VA data and may not be comparable across regions, because it relies on the views of local doctors about the likely causes of death. In addition, although several studies have suggested that PCVA is reasonably accurate, such studies have usually included information collected from household members about medical records or contacts with health services. In regions where there is little contact with health services, PCVA may be much more inaccurate. Ideally what is needed is a method for assigning causes of death from VA data that does not involve physician review. In this study, the researchers have developed a statistical method—the symptom pattern (SP) method—for analyzing VA data and asked whether it can overcome the limitations of PCVA.

**What Did the Researchers Do and Find?:**

The SP method uses VA data collected about a group of patients for whom the true cause of death is known to calculate the probability for each cause of death that a household member will answer yes when asked about various symptoms. These so-called “symptom properties” can be used to calculate population cause-specific mortality fractions (CSMFs—the proportion of the population that dies from each disease) from VA data and, using a type of statistical analysis called Bayesian statistics, can be used to assign causes of deaths to individuals. When used with data from a VA study done in China, the SP method estimated population CSMFs with an average relative error of 16% (this measure indicates how much the estimated and true CSMFs deviate), whereas PCVA estimated them with an average relative error of 27%. At the individual level, the SP method assigned the correct cause of death in 83% of cases; PCVA was right only 69% of the time. Removing the medical record recall section of the VA data had little effect on the accuracy with which the two methods estimated population CSMFs. However, whereas the SP method still assigned the correct cause of death in 78% of individual cases, the PVCA did so in only 38% of cases

**What Do These Findings Mean?:**

These findings suggest that the SP method for analyzing VA data can outperform PCVA at both the population and the individual level. In particular, the SP method may be much better than PCVA at assigning the cause of death for individuals who have had little contact with health services before dying, a common situation in the poorest regions of world. The SP method needs to be validated using data from other parts of the world and also needs to be tested in multi-country validation studies to build up information about how culture and language affect the likelihood of specific symptoms being reported in VAs for each cause of death. Provided the SP method works as well in other countries as it apparently does in China, its adoption, together with improvements in how VA data are collected, has the potential to improve the accuracy of cause-of-death data in developing countries.

**Additional Information.:**

Please access these Web sites via the online version of this summary at http://dx.doi.org/10.1371/journal.pmed.0040327.

• An accompanying paper by Murray and colleagues describes an alternative approach to collecting accurate cause-of-death data in developing countries

• World Health Organization provides information on health statistics and health information systems, on the International Classification of Diseases, on the Health Metrics Network, a global collaboration focused on improving sources of vital statistics and cause-of-death data, and on verbal autopsy standards

• Grand Challenges in Global Health provides information on research into better ways for developing countries to measure their health status

## Introduction

Cause of death data are a critical input into debates on public health, assessment of epidemiological patterns and trends, and guidance on allocation of scarce resources for pubic health and medical care [1]. Cause of death data for these purposes are equally important for high-income and low-income countries, but the challenge of comparable measurement is different. For high-income countries, the main task is to minimize systematic differences in assignment of causes of death over time and across populations. For developing countries, where vital registration systems are generally not complete, and progress toward higher levels of coverage and certification has been unacceptably slow, the challenge is how to make better use of alternative cause-of-death measurement approaches to reliably estimate population-level mortality patterns. This paper presents a new method for assigning causes of death based on an interview of members of the decedent's household.

Considerable effort has been invested to assess mortality by cause based on information collected after death from household members—termed since the early 1980s “verbal autopsy.” Use of this approach can be traced to 1931 [2], but the first large-scale systematic application of it was in Matlab, Bangladesh [3]. Because vital registration coverage has not significantly improved in developing countries, interest in verbal autopsy (VA) methods has expanded to at least four distinct settings: clinical trials and large-scale epidemiological studies; demographic surveillance systems, such as those in the INDEPTH network [4]; national sample surveillance systems [5,6]; and household surveys [2]. Despite increasing and widespread use of VA in field data collection, utilization of VA data for national epidemiological monitoring and global/regional burden of disease estimation has so far been limited. For example, WHO makes almost no use of VA data for adult cause of death estimation because of the heterogeneity of VA implementation and non-ICD (International Classification of Diseases) cause lists. A notable exception has been the use of VA data (for under-age-5 cause of death) to dramatically reduce global estimates of measles mortality in children [7].

The heterogeneity of VA design and implementation spans several critical issues: instrument design, method for assigning underlying cause of death, cause of death lists, recall period, and type of respondent [8]. Two are particularly critical: instrument design and method for cause-of-death assignment. First, the instrument used to collect data after death from a household member varies widely, limiting comparability. Some field sites use open-ended questions in an unstructured interview, while other instruments are highly structured checklists of symptoms and signs. Most instruments also elicit information on health-care use, medical diagnoses, and documentation of the cause of death. Second, data collected through the VA instrument are analyzed to assign an underlying cause of death in one of three ways: physician review, expert algorithms, and statistical models. Physician review is the dominant approach, since the critical review of signs and symptoms approximates the clinical review of medical records and case histories that informs death certification in hospitals and other health-care settings. In addition, physician review has been the dominant approach because convincing alternatives have not been widely available. Most sites use more than one physician to review the VA responses and assign an underlying cause of death [8]. The use of physician review has served to mask the heterogeneity of VA instruments and content, as studies simply report the results of the physician assignment of causes of death. Validation studies have been published showing high sensitivity and specificity for selected causes [9–11], but even with high sensitivity and specificity, there are challenges in using physician-coded VA (PCVA) to derive unbiased estimates of population cause-specific mortality fractions [12–15].

Physician coding of VA data limits the potential use of VA for population heath monitoring in three important ways. First, local physician views on population epidemiology and causal hierarchies probably exert a profound influence on the diagnoses recorded. The influence of local medical culture is also seen in vital registration data with medical certification [16]. Strong views of local physicians will decrease the comparability of PCVA across populations. Differences in estimated cause-specific mortality rates may not be due to differences in the VA data themselves but simply because of differences in these local views. Second, the sensitivity and specificity of PCVA for deaths out of hospital may be much lower than currently recognized. Nearly all published validation studies have included collection of information from the household on medical records, death certificates, and recall of the cause of death by a health-care worker. Inclusion of this information in the VA will clearly exaggerate the performance of PCVA for deaths outside of hospital. Third, PCVA substantially increases the time and cost required to analyze VA data, limiting large-scale population applications.

There are three broad research directions underway to create more standardized underlying cause of death assignment using VA: creation of consensus standardized VA instruments; development of expert algorithms for assigning the underlying cause of death; and development of statistical methods. First, several initiatives are creating and testing standardized instruments for neonatal, child, and adult deaths [4,17]. Second, expert algorithms are based on the concept of distilling the process of physician review into standardized rules. These are meant to systematize the logic that an individual physician would use to assign a cause of death, but with the added advantage of consistent application [18,19].

The third approach is to develop statistical models to predict cause of death based on the detailed results of a standardized VA instrument [19–23]. A wide range of modeling strategies have been used, including logistic regression, neural networks, and Bayesian approaches. Byass et al. [21,24] have argued for the application of Bayes's theorem to individual cause-of-death assignments by using the symptom-level data recorded in the VA. Using data for rural Vietnam, that work demonstrated that such an approach has promise in categorizing individual causes of death when compared to PCVA. Recently, King and Lu [25] presented a sophisticated method for estimating cause-specific mortality fractions directly without individual cause-of-death attribution. Their method resolves the problem of generalizing VA analysis to the population based on test properties quantified in hospital validation studies. In this paper, we bring together the insights from King and Lu, and Byass et al. into a single method, which we have termed the symptom pattern method (SP), to estimate both population-level cause-specific mortality fractions (CSMFs) and underlying causes of death for individuals. Using data from China, we investigate the performance of this method in comparison to PCVA at the population level and at the individual death level.

## Methods

From a validation cause-of-death dataset for each variable included in the VA instrument (henceforth called symptoms), we can calculate the probability of household members responding in each response category for each cause of death. To simplify the explanation, let us assume all items in the VA instrument are dichotomous. Let *S_ij_* be the probability that household members say yes to symptom *i* for cause of death *j*. If one considers symptom *i* like a diagnostic test, *S_ij_* is the sensitivity of symptom *i* for disease *j*. Note that unlike most of the literature on VA, we are not referring to the sensitivity of the entire VA instrument subject to physician review, but rather to sensitivity of a single symptom in the instrument.

Let *P_j_* be the CSMF for cause *j*, and *S_1_* be the proportion of all VA responses that say yes to symptom 1 in the population. Since the *k* causes of death are mutually exclusive and collectively exhaustive, the fraction of deaths that reported yes to symptom 1, *S*_1_, must be the sum of the fraction of deaths that reported yes to symptom 1 and died of cause 1 (*S*_11_), the fraction of deaths that reported yes to symptom 1 and died of cause 2 (*S*_12_), and so on. This gives us

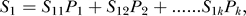



Since each death is in one and only one of the cause groups, it must also be true that

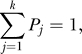



and it then follows that

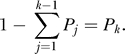



Combining the relationships above, we see that for any set of *k* − 1 symptoms, the following set of linear equations must hold:

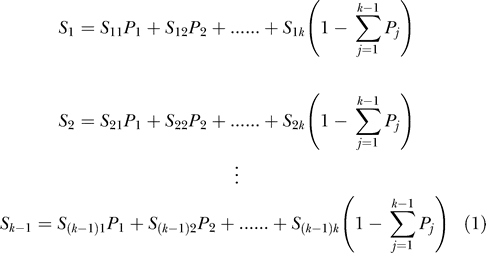



If the *S_ij_* are known, this system has *k* − 1 equations and can be solved for the *k* − 1 unknown values of *P_1_* through *P_k_*_ − 1_. If we have more than *k* − 1 symptoms, or in the presence of measurement error, we could also estimate *P_1_* through *P_k_*_ − 1_ using regression techniques.

We wish to employ these equations to estimate the CSMFs, *P_1_* through *P_k_*_ − 1_. A first pass at this problem might select a random subset of *k* − 1 symptoms and obtain values for *P_j_* by estimating a regression, or by solving the system of equations. This process could be repeated, drawing many sets of *k* − 1 symptoms, resulting in many estimates of *P_j_*. A final estimate for each *P_j_* could then be obtained by computing the mean of the estimates gathered over all of the symptom draws. King and Lu advance this basic approach by randomly selecting sets of symptoms, called symptom profiles, and computing the *S_i_* and *S_ij_* values in the system of equations above for the symptom profiles, rather than for the individual symptoms [25]. This relaxes the assumption that, within a true cause of death group, the responses to individual symptoms are completely independent of one another. Over multiple draws of symptom profiles, then, King and Lu's strategy accounts for the correlation between groups of symptoms. Each random draw of symptom profiles yields an estimated set of *P_j_* values. The mean of these *P_j_* estimates over all of the iterations is then used as the best estimate of the CSMF for each cause *j*. We ran the algorithm with 300 draws of symptom profiles. We configured it to select 16 symptoms, each chosen from the set of symptoms with equal probability; the symptom profiles were then the combinations of those 16 symptoms that actually appeared in the data.

After obtaining CSMF estimates, we can use them as an input in a strategy to assign causes to individual deaths. For an individual death *i,* the probability of dying from cause *j* can be expressed following Bayes's theorem as:

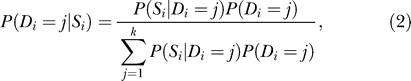
where *P*(*D*_i_ = *j*|*S*_i_) is the probability of individual *i* dying from cause *j*, conditional on the observed vector of symptom responses, *S_i_*. In the terminology of Bayes's theorem, this is called the posterior. It can be calculated for each individual and cause of death. Since the causes of death are mutually exclusive and collectively exhaustive, the sum of these posterior probabilities for any individual death will be 100%. *P*(*S*_i_|*D*_i_ = *j*) is the probability of observing the vector of symptoms *S_i_* conditional on individual *i* having died from cause *j*. If symptoms are largely based on the biology and natural history of the disease, this probability can be determined from well-designed studies in which a gold standard diagnosis is known. The expression *P*(*D*_i_ = *j*), known as the prior, is the probability that individual *i* died of cause *j*, without taking into account the symptom response data. In this case, we use the CSMFs estimated with King and Lu's strategy, outlined in the discussion above, as the prior for individual-level cause of death assignment. In the same way that King and Lu use draws of symptom profiles to estimate the CSMFs, we repeatedly draw random combinations of symptoms, compute *P*(*S*_i_|*D*_i_ = *j*), and estimate posterior probabilities for each cause of death. We repeat this many times and take the mean of the results as our final estimate for the posterior probability for each cause. In the results we present here, we choose symptom profiles by selecting 15 symptoms from the entire set, with each symptom equally likely to be chosen. The symptom profiles are then the observed combinations of those 15 symptoms that occur in the data. We draw symptom profiles 50 times. In order to uniquely assign an underlying cause of death to compute concordance rates and to compare to PCVA, for each individual, our cause-of-death estimate is then the cause that has the highest estimated posterior probability.

We compute population CSMFs two ways. First, we take the posterior probabilities estimated for each individual—that is, the output of the individual-level application of Bayes's theorem—and we aggregate these posterior probabilities by cause. We then use the relative distribution of posterior probabilities for each cause of death, across all of the individuals, as our updated estimate of the CSMFs. Second, we sum the number of deaths assigned to each cause based on which cause has the highest probability. The advantage of the first approach is that it more directly reflects the information content in the data about the presence of each cause in the population. The advantage of the second approach is that it is fully consistent with the assignment of a unique cause of death at the individual level. We present results from the first strategy and evidence that shows that the two strategies yield very similar results.

To illustrate this approach, we have used data from our China VA validation study based on medical record review [11]. For the 2,089 deaths with a gold standard underlying cause of death, and a completed VA instrument, we have divided deaths into 23 mutually exclusive and collectively exhaustive cause groups (see Table 1). These groups have been selected such that there were at least 15 deaths in each cause group, including the combined ‘other' groups. Based on the VA instrument, 47 dichotomous symptom variables have been selected. We have first tested the validity of our SP method by sampling with replacement the 2,089 deaths to generate two datasets: a validation dataset of 4,600 deaths, 200 for each cause, to estimate the values of *S_ij_*, and a community dataset of 10,000 deaths, to which we apply the SP method. One limitation of our use of simulation datasets is that single deaths from the original dataset may be sampled multiple times, resulting in less variation in symptom profiles than we would expect to see in real data.

**Table 1:**
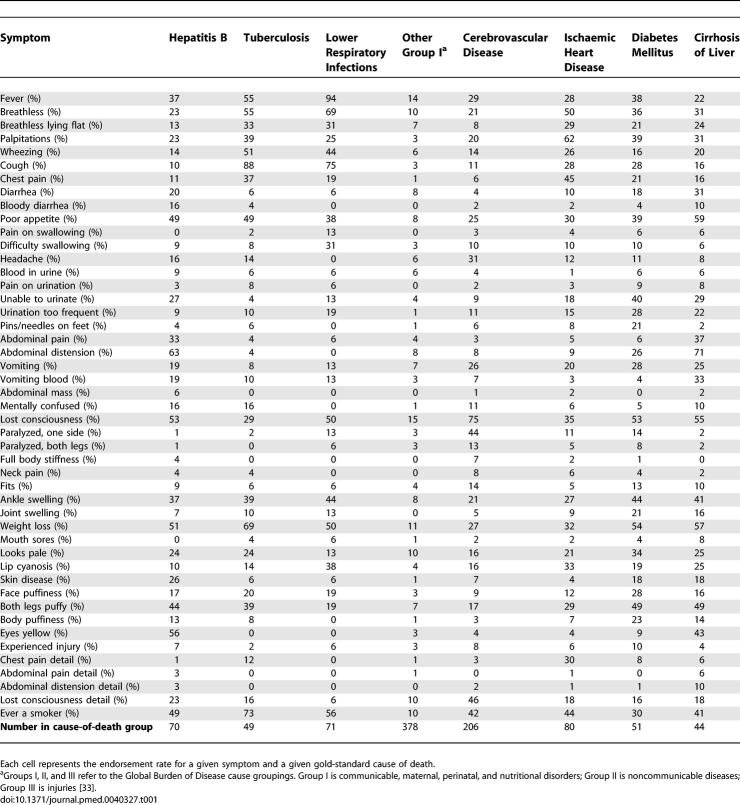
Verbal Autopsy Symptom Endorsement Rates by Cause-of-Death Group

**Table 1:**
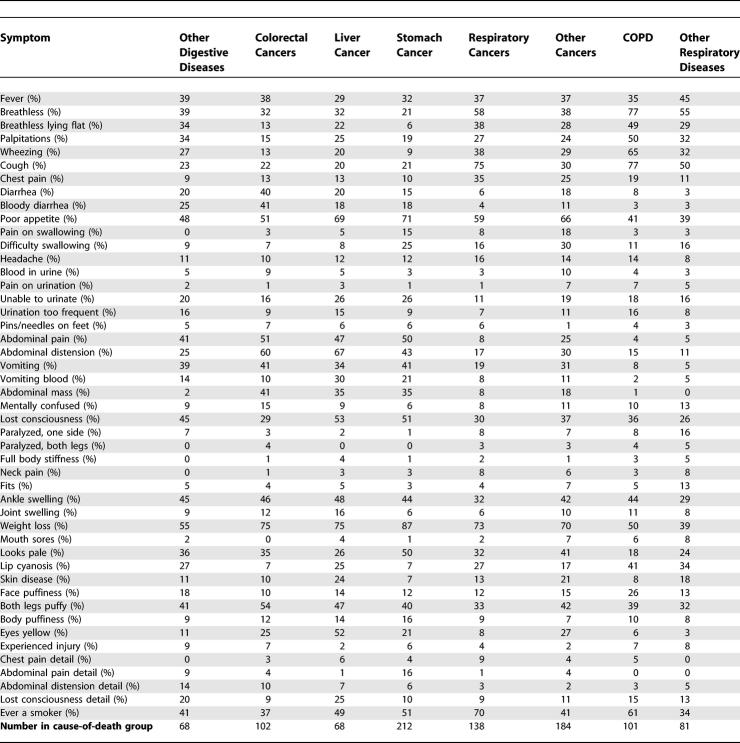
Extended.

**Table 1:**
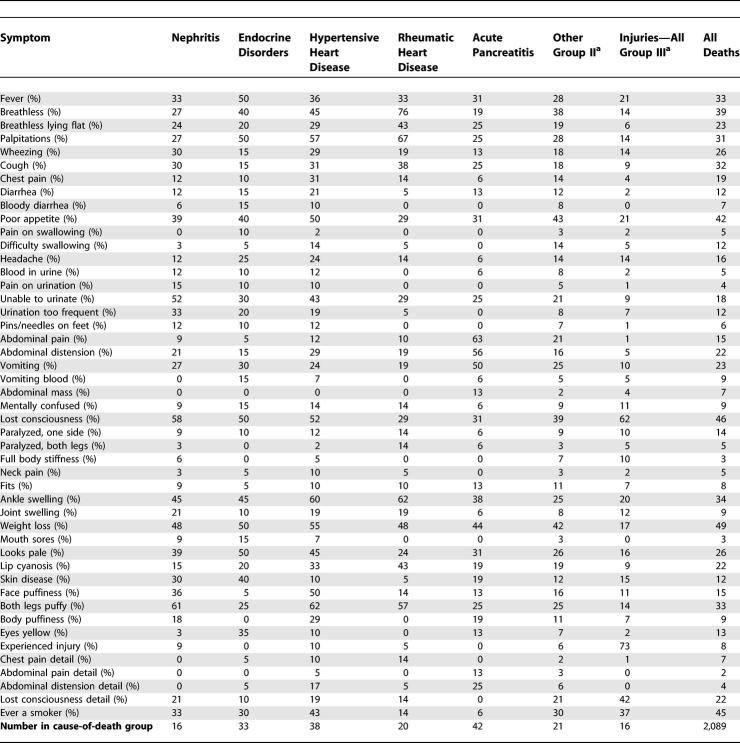
Extended.

The gold standard underlying cause of death was derived through expert review of the medical records by a panel of three physicians. Medical records were abstracted by trained personnel including clinical information, diagnostic results, and treatment details. Expert review death certificates were coded by the World Health Organization (WHO) Collaborating Centre for Classification of Diseases in Beijing to select and code the underlying cause of death according to the International Classification of Diseases, Tenth Revision (ICD-10) [11,26]. Further information is available in Rao et al. [27].

As in most VA studies that use PCVA, the instrument used in China also collected information on medical record recall from the household. This information includes items on cause of death from the death certificate and burial permit, hospital forms, and the child's health card. Information from at least one of these sources was available for 94% percent of the deaths. We have repeated our analysis using both the full VA data and the medical record history data by treating the recall of medical records as if they were simply more *S_ij_* values. To provide a comparison to PCVA, two Chinese physicians recoded the 2,089 VAs blinded to medical record recall. This empirical work also provides a test of the importance of medical record recall on PCVA as opposed to information contained in the symptom recall.

To further test the sensitivity of the SP method to the cause composition of mortality in a community, we have used sampling with replacement of the 2,089 China validation dataset deaths with weights by cause to alter the background cause composition for the community sample. We have used weights based on the WHO estimates of cause of death composition for South East Asia subregion D (SEAR-D; primarily India) and Western Pacific subregion B (WPR-B; primarily China). For the same community samples, we have calculated the same results for PCVA as well as for the SP method.

Three metrics of predictive validity have been computed: for the population-level results, we calculate the average percent relative error and the average absolute error for the 23 CSMFs. The average percent relative error is the average amount that the estimated CSMF deviates from the true CSMF, as a fraction of the true CSMF. In other words, it is the average, over all of the causes, of the quantity

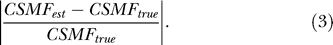



The average absolute error is the average absolute amountthat the estimated CSMF deviates from the true CSMF, over all of the causes of death. In other words, it is the average, over all of the causes, of the quantity

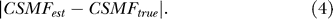



Performance of the individual cause of death assignment has been evaluated using the concordance rate: the percentage of times that the true cause, based on the hospital record review, has been correctly assigned. Because this step produces a probability distribution across causes, we calculate the concordance rate based on the cause of death assigned the highest probability of death.

## Results

Table 1 provides the *S_ij_* values from the China VA study for the 47 main symptoms. We have excluded from this analysis information on the duration of symptoms. We undertook extensive analysis using symptom duration data as further *S_ij_* values, but this did not improve the performance of the method over and above inclusion of just the symptoms. We can think of each entry in the table as the properties of the symptom (row) for the cause of death (column). For example, the entry at the fifth row and third column is 44%, meaning that 44% of VA respondents for deaths from lower respiratory infections responded yes when asked if the deceased wheezed. There is clearly considerable information content in the individual symptoms even without information on the sequencing, duration, and clustering of symptoms. There is also clear evidence that, like any survey instrument, there is a significant background noise for each symptom. Injuries, whose occurrence can be expected to be uncorrelated with many symptoms, provide insight into these background rates. For example, among deaths due to injuries, 21% of household members reported fever, 21% poor appetite, 17% weight loss, and 15% skin disease; only 73% reported that the decedent suffered an injury. The latter surprising finding has also been observed in South Africa [28]. Some symptoms, which medical knowledge would suggest are highly specific such as paralysis on one side, are present for more than 10% of decedents for seven of 27 causes, suggesting that this symptom has a different cultural interpretation in China.

Table 2 summarizes the information from the last part of the VA instrument on the recall of causes of death in the death certificate, burial certificate, and household recall. We have combined the data from all these sources into a single measure, as they all reflect information from providers or government registries as reflected through the recall and experience of household members. These symptoms are our coding of the information that physicians with access to medical record recall had; each symptom is a phrase that appears in the medical records, on the death certificate, or on the burial certificate. We identified key phrases used in the medical records, death certificates, and burial certificates and mapped these phrases to ICD categories. It is possible that meaningful local phrases may have been missed in this mapping, which would tend to bias our analysis against the SP method. The physicians who were blinded to medical record recall did not have access to this information, and our results without medical record recall do not make use of it. These can be interpreted in the same way as Table 1. So, an endorsement rate of 4% for the third row (lung cancer) and second column (TB) means that for 4% of deaths whose underlying cause was TB, the medical records, burial certificate, or death certificate mentioned lung cancer.

**Table 2:**
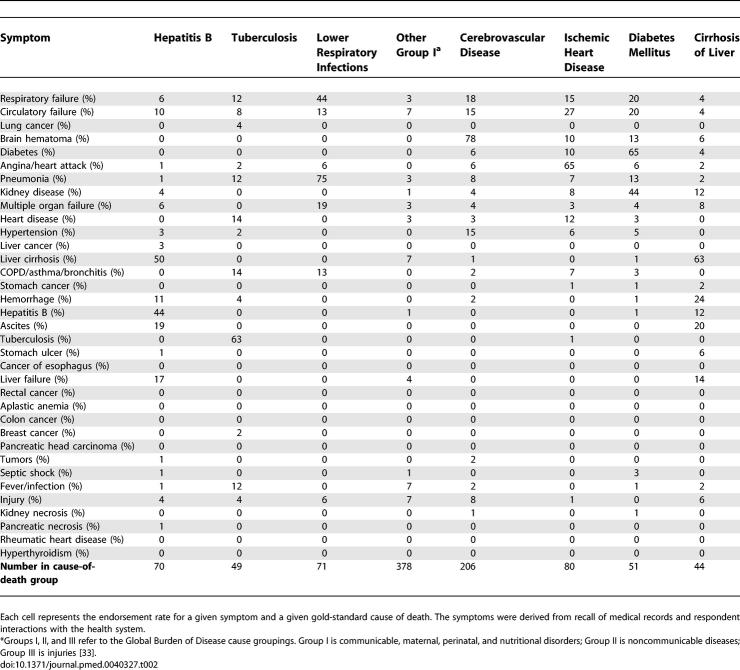
Verbal Autopsy Medical Record Recall (MRR) Symptom Endorsement Rates by Cause-of-Death Group

**Table 2:**
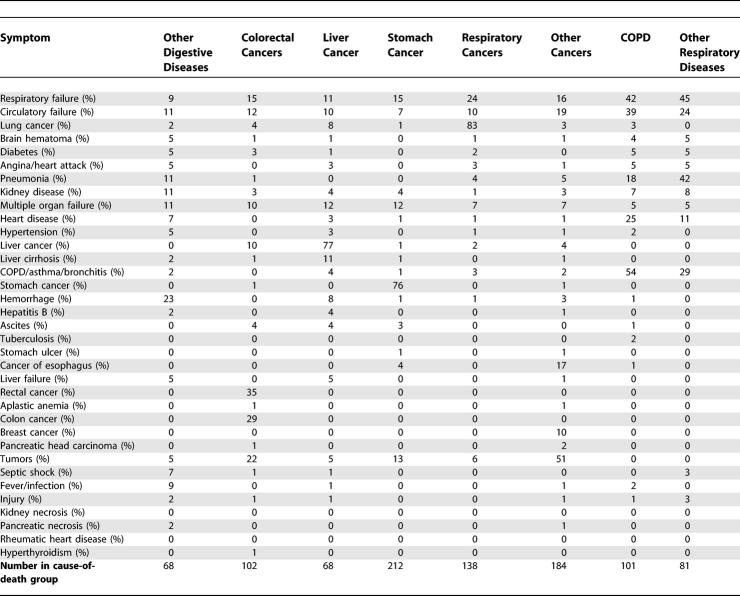
Extended.

**Table 2:**
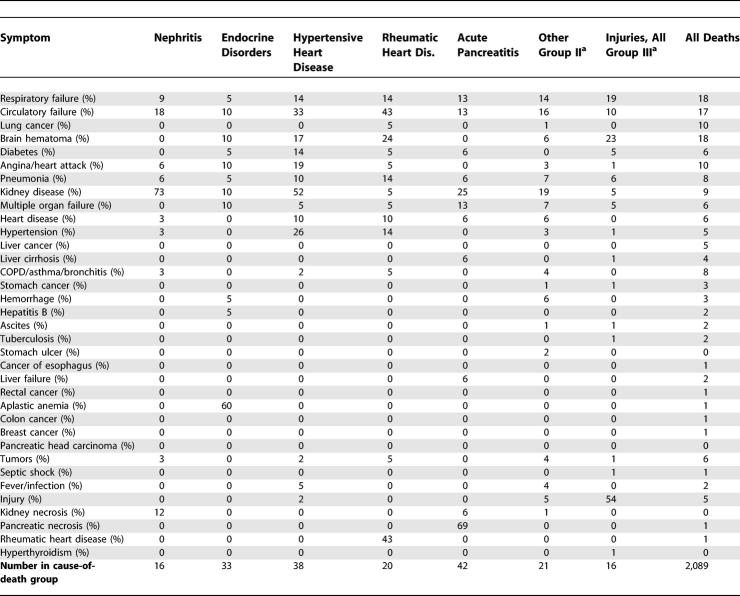
Extended.

**Figure 1:**
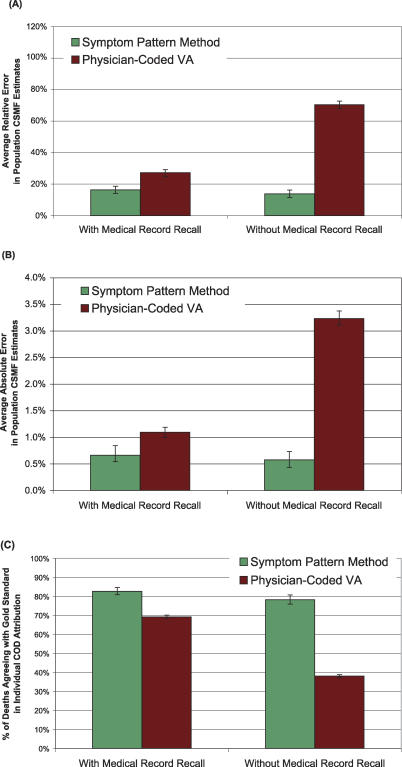
Summary Measures of Error in Population and Individual-Level Cause-of-Death Estimates for China Data Results are from the SP method and PCVA with and without the symptoms derived from recall of medical records. (A) Average relative error in population-level CSMF estimates for China data. (B) Average absolute error in population-level CSMF estimates for China data. (C) Percent concordance (percent of deaths agreeing with gold standard) for individual-level cause-of-death (COD) assignments in China data.

Figure 1 summarizes the application of the SP method and PCVA to a 10,000 community sample of deaths in China. With medical record recall, the SP method estimates the true CSMFs with average relative error of about 16%, while PCVA gives relative error of about 27%. In absolute terms, the average difference between the estimated and the true CSMF is 0.7% for the SP method, and 1.1% for PCVA. At the individual level, PCVA correctly assigns the cause of death in slightly more than two-thirds of the cases, while the SP method is correct for over 80% of the deaths. The dependence of the physician assignment of cause of death based on the recall of medical records is evident in the dramatic change in PCVA performance when the medical records are excluded. The average relative error for the CSMFs increases to over 70% and the concordance rate at the individual death level drops to below 40%. The SP method estimates of the CSMFs is unaffected by the exclusion of the medical record recall; the relative error remains at about the same level, 14%, and the absolute error, at 0.6%, is similar as well. The individual concordance rate drops slightly, to just under 80% which is still significantly better than PCVA. But the reality is that symptoms alone are probably not a sufficient basis for assigning a unique cause of death to each individual; diagnostic tests and imaging studies are critical for diagnosis in many cases.

**Figure 2:**
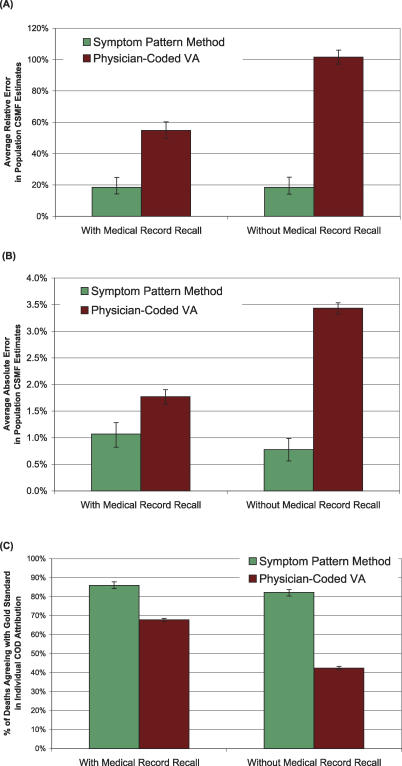
Summary Measures of Error in Population and Individual-Level Cause-of-Death Estimates, Background Cause Composition of Mortality Changed to Simulate Distribution in WHO Subregion SEAR-D Results are from the SP method and PCVA, with and without the symptoms derived from medical record recall. (A) Average relative error in population-level CSMF estimates for SEAR-D. (B) Average absolute error in population-level CSMF estimates for simulated SEAR-D data. (C) Percent concordance (percent of deaths agreeing with gold standard) for individual-level cause of death (COD) assignments in SEAR-D data.

**Figure 3:**
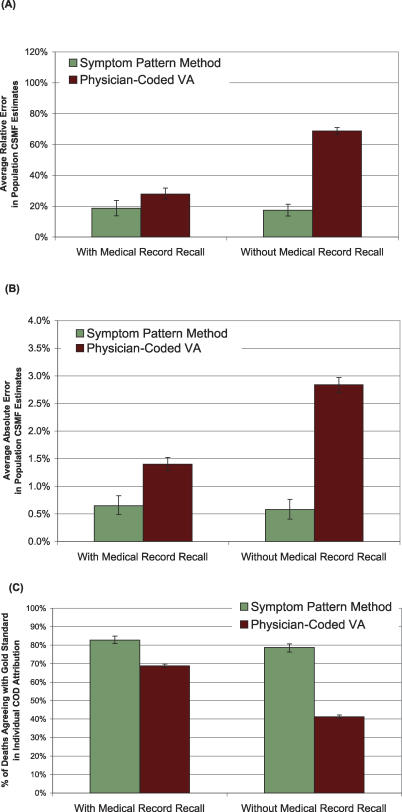
Summary Measures of Error in Population and Individual-Level Cause-of-Death Estimates, Background Cause Composition of Mortality Changed to Simulate Distribution in WHO Subregion WPR-B Results are from the SP method and PCVA, with and without the symptoms derived from medical record recall. (A) Average relative error in population-level CSMF estimates for WPR-B. (B) Average absolute error in population-level CSMF estimates for simulated WPR-B data. (C) Percent concordance (percent of deaths agreeing with gold standard) for individual-level cause of death assignments in WPR-B data.

A long-standing concern with VA validation studies has been that sensitivity and specificity of PCVA may vary as a function of the cause composition of mortality [12,29]. To explore this problem, we have varied the background cause composition of the community sample of deaths generated through sampling with replacement with cause of death composition weights that match the SEAR-D and WPR-B WHO subregions. Figures 2 and 3 summarize the three measures of predictive validity: CSMF average relative error, CSMF average absolute error, and individual concordance rates. The results for both regions demonstrate that the SP method produces quite accurate estimates of CSMFs and individual concordance rates that are over 75% with no medical record recall and over 80% with medical record recall. At least based on these two simulation studies, there is no evidence that the SP method is sensitive to changing the cause composition of mortality.

Table 3 compares individual concordance rates for the China community simulated sample by cause of death with and without medical records for PCVA and the SP method. For nine of the causes of death, the concordance rates for physicians drops by more than 75% including: hepatitis B, diabetes, colorectal cancer, stomach cancer, lower respiratory infections, hypertensive heart disease, endocrine disorders, rheumatic heart disease, and acute pancreatitis. For some causes, such as hepatitis B and acute pancreatitis, PCVA correctly assigns no deaths when medical record recall is removed. For the SP method, the lowest concordances are in other group I conditions, at 34%, other group II conditions, at 61%, and lower respiratory infections, at 65%. We would generally expect SP to perform least well on residual categories of death like other group I conditions. These residual categories represent an average symptom profile of distinct diseases included in the residual. Much of the distinct information content in the set of *S_ij_* values is thus lost due to averaging across different entities. The poor results for lower respiratory infections may be due to the small number of cases, 16, in the original validation dataset. Without medical record recall, the same three conditions perform worst, at 53%, 64%, and 70% respectively.

**Table 3:**
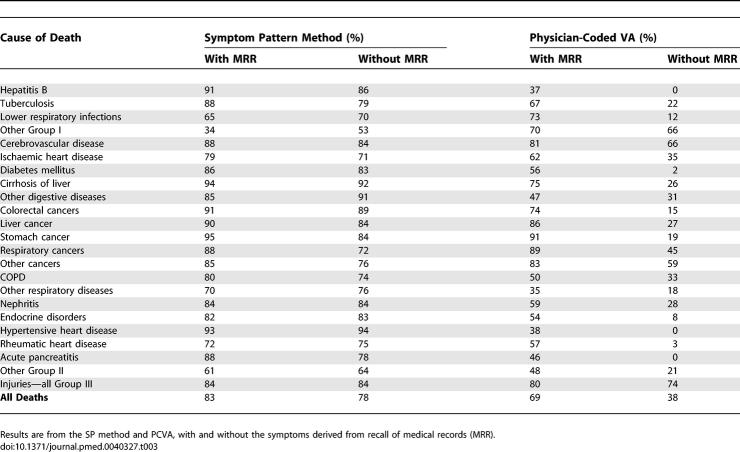
Individual Concordance (Percent Agreement with Gold Standard Cause of Death), by Gold Standard Cause of Death in China Community Simulated Sample

**Figure 4:**
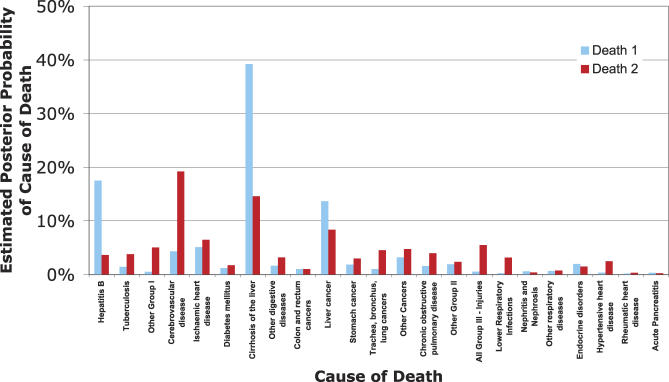
Sample Posterior Cause-of-Death Probabilities Produced by the Symptom Pattern Method for Two Deaths Whose True Cause Was Cirrhosis of the Liver The height of each bar over each cause of death represents the probability, estimated using the SP method, of the individual having died of that cause.

Figure 4 gives an example of the posterior distributions produced by SP for two deaths in one of the simulated datasets. The gold-standard cause of death for both of these is cirrhosis of the liver. Since we assigned the most probable single cause to each death, we can see that SP would correctly assign the cause in the first death, where cirrhosis is the most probable posterior cause, while SP would be incorrect for the second death, where cerebrovascular disease is most probable in the posterior. The distribution for the second death is spread across the causes more evenly, meaning that the symptoms were not as predictive as they were for the first death, where they are more focused on cirrhosis, hepatitis B, and liver cancer. Although we have only used the most probable posterior cause in assigning a single cause to each death, these posterior distributions have information that could be exploited for other purposes. For example, the diffuseness of the distribution gives us some idea of the certainty of the cause assignment.

**Figure 5:**
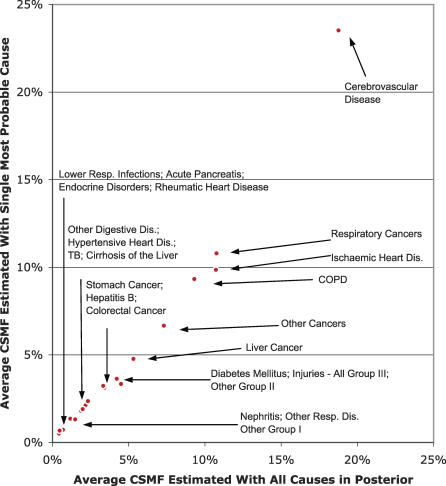
Comparison of the Average CSMF Estimated Using the Single Most Probable Posterior Cause and the Average CSMF Estimated Using the Posterior Distribution of All 23 Causes The means were computed over the 100 draws of the China data.

As noted in the Methods section, we can calculate population CSMFs by summing the number of deaths assigned to each cause based on the highest probability cause of death in the posterior distribution or we can sum the posterior probabilities for each death across each cause. Figure 5 compares these two approaches for the 23 causes in the China data. For each cause, it shows the average CSMF estimated for the 100 draws of data obtained by using the single highest probability cause to the CSMF obtained by using the full posterior distribution. The results are very similar, with a correlation of 0.98. The most noticeable outlier is the CSMF for cerebrovascular disease, which is generally estimated to be larger when the single most probable cause is used.

## Discussion

Overall, the SP method does remarkably well. At the population level, it provides more accurate estimates of cause-specific mortality fractions than PCVA; indeed, at least in China, PCVA is not a viable method for estimating the cause composition of mortality at the population level. At the individual level, it clearly outperforms PCVA in assigning causes of death to individual responses, using the same information. The difference is most striking when the two strategies are not allowed access to information from household recall of medical records, which is the test most relevant to implementing verbal autopsy in a resource-poor setting. Based on this analysis of data from China, the superiority of the SP method for estimating CSMFs and assigning causes of death at the individual level does not appear to be affected by changing the background cause of death composition in the population sample. In contrast, in China PCVA appears to be extremely sensitive to the availability of household recall of medical records.

Cause of death assignment for some deaths must be highly uncertain, because the information content in signs and symptoms is limited. While we have emphasized in presenting results the comparison of PCVA with SP using the highest-probability cause for each death, this method generates an uncertainty distribution across causes, as illustrated for two deaths in Figure 4. These distributions are a direct reflection of the information content in the VA responses. Future studies may be able to make direct use of this information to guide a deeper recognition of which causes can be more accurately identified on the basis of signs and symptoms alone. Conventions may be developed such that deaths for which the uncertainty distribution is diffuse across many causes would be assigned to an unknown or ill-defined category. In any case, the SP method provides a more realistic reflection of the information content in the VA dataset.

Reanalysis of the China VA validation data by physicians blinded to medical record recall shows that physicians are highly influenced by this information, as one might expect. Most published validation studies on PCVA include medical record recall. The levels of sensitivity, specificity, or overall concordance rates reported in the literature for deaths with medical record recall are substantially overstated for deaths that occur without contact with medical services. Some authors [5] have even claimed that high inter-rater reliability between physicians is evidence of the validity of PCVA. Of course, high inter-rater reliability could simply be due to multiple physicians using the medical record recall to assign the cause of death, not the symptom patterns. As such, inter-rater reliability provides no direct evidence of validity.

Because the SP method does not use physicians in assigning causes of death, it has the potential to dramatically reduce the cost of VA at the population level and to increase the comparability of results across populations. Use of VA in routine public health surveillance has in part been limited by the availability of standardized international instruments but more so by the practicality of using physicians trained in the review of VA data. The SP method requires no individual coders and will reduce cost and implementation issues. Another major advantage is that it can be implemented in a completely replicable manner in different populations. By standardizing how causes of death are assigned and removing the unquantifiable influence of physician prior beliefs from the results, comparability across populations within a country and across countries will be substantially enhanced.

The critical requirement for our method is that we know the set of symptom level sensitivities *S_ij_* values or that we can adequately predict these sensitivities for a population. This is an important assumption with several implications. First, we need to measure the *S_ij_* values in a number of populations and to measure them for as many detailed causes as possible. More data on stroke *S_ij_* values are not likely to be very useful, but more for diseases such as hepatitis B or COPD would be. Measuring the *S_ij_* values may also be challenging in resource-poor settings where deaths coded to a reliable gold standard are difficult to locate using existing hospital medical records. To address this issue ongoing field studies, in Tanzania, India, and the Philippines, are defining more carefully strict diagnostic criteria for gold standard cause attribution. Second, we need to study the variability of the *S_ij_* values as a function of culture, individual respondent characteristics such as income, education or medical knowledge, and decedent characteristics such as the experience of health care. Large validation datasets such as the one being collected in the Grand Challenges in Global Health 13 project [30] may provide an opportunity to study the predictors of the *S_ij_* values. Third, symptom responses may be profoundly affected by disease natural history but they may also potentially be affected by cultural factors; that is, it may be that the items on the VA questionnaire will behave differently as a result of culture. In these situations, local validation studies will be needed to define how a VA item functions in a given population.

New data are needed in different populations to confirm the success of the SP method. In this study, we simulated data for the Chinese population and two WHO subregions based on a sample of VA responses from China; further data collection will allow us to test the generalizability of these results to other parts of the world.

We suggest that VA validation studies need to be radically redesigned. Most importantly, samples need to be chosen so as to get approximately equal numbers for important causes of death rather than a random sample of the population if we want to accurately characterize *S_ij_*. This is an important reversal of the usual validation study in which a sequential sample of deaths in hospital is often used. As noted, stricter review processes to determine the gold standard diagnosis are also needed, so that only cases with medical records in which the underlying cause can be determined with substantial certainty are included. Such stricter gold standards are more feasible to implement in low-resource settings when the number of deaths meeting the gold standard for each cause can be optimized.

In most cases, validation studies are undertaken for deaths that have occurred in hospital, and household recall of the hospital experience is included in what is presented to the physicians. The key exception to this has been validation studies of VA that seek to categorize deaths into only two causes, AIDS and non-AIDS, where HIV serostatus in a cohort is used as a the gold standard [27,31,32]. When this information is excluded from the analysis, our CSMFs are still as accurate but individual cause of death concordance drops. In other words, VA at the individual level may not work as well as published validation studies suggest when applied to deaths of individuals who did not have contact with health services prior to death. This is not a limitation of PCVA or of our approach. Rather, it may simply be a reflection of the true information content in the symptom recall. Further research studies, particularly on PCVA excluding all hospital and death record information, will help to better understand this effect.

Our approach to VA cause of death assignment should be tested and validated in other populations outside of China. This will be greatly facilitated if research teams are encouraged to put the full microdata from validation studies into the public domain. We also see a need for a multi-country validation exercise and a series of cause-of-death surveys to derive *S_ij_* data, possibly using the newly developed WHO standardized tools. If our methods are confirmed in other populations, the cost of obtaining useful, comparable cause of death information in poor countries will be greatly reduced. Combined with advances being made on verbal autopsy instrument design, this research has the potential to rapidly improve knowledge about countries' epidemiological priorities that would otherwise be impossible from the slow progress of vital registration in developing countries.

## Abbreviations

CSMF: cause-specific mortality fraction
ICD: International Classification of Diseases
PCVA: physician-coded verbal autopsy
SEAR-D: WHO South East Asia subregion D
SP: symptom property method
VA: verbal autopsy
WHO: World Health Organization
WPR-B: WHO Western Pacific subregion B
